# Mapping Health Fragility and Vulnerability in Air Pollution–Monitoring Networks in Dallas–Fort Worth

**DOI:** 10.3390/ijerph20031807

**Published:** 2023-01-18

**Authors:** Kari Northeim, Joseph R. Oppong

**Affiliations:** 1 Department of Biostatistics and Epidemiology, School of Public Health, University of North Texas Health Sciences Center, 3500 Camp Bowie Blvd, Fort Worth, TX 76104, USA; 2 Department of Geography and the Environment, University of North Texas, 1704 W. Mulberry, Denton, TX 76203, USA

**Keywords:** air pollution, climate change, vulnerable populations

## Abstract

Environmental air pollution remains a major contributor to negative health outcomes and mortality, but the relationship between socially vulnerable populations and air pollution is not well understood. Although air pollution potentially affects everyone, the combination of underlying health, socioeconomic, and demographic factors exacerbate the impact for socially vulnerable population groups, and the United States Clean Air Act (CAA) describes an obligation to protect these populations. This paper seeks to understand how air pollution monitor placement strategies and policy may neglect social vulnerabilities and therefore potentially underestimate exposure burdens in vulnerable populations. Multivariate logistic regression models were used to assess the association between being in an ozone-monitored area or not on 15 vulnerability indicators. It was found that the odds of not being in an ozone-monitored area (not covered, outside) increased for the predictor mobile homes (OR = 4.831, 95% CI [2.500–9.338] and OR = 8.066, 95% CI [4.390–14.820] for the 10 and 20 km spatial units, respectively) and decreased for the predictor multiunit structures (OR = 0.281, 95% CI [0.281–0.548] and OR = 0.130, 95% CI [0.037, 0.457] for the 10 and 20 km spatial units, respectively) and the predictor speaks English “less than well” (OR = 0.521, 95% CI [0.292–0.931] for 10 km). These results indicate that existing pollution sensor coverage may neglect areas with concentrations of highly vulnerable populations in mobile homes, and future monitoring placement policy decisions must work to address this imbalance.

## 1. Introduction

Over seven million people die annually from the global problem of air pollution [1]. Ozone air pollution has both health and environmental effects and is of particular interest because it damages agriculture, harms respiratory functions, and contributes to climate change [2,3,4,5,6]. Higher concentrations of air pollutants occur in urban environments, driven primarily by the energy and transportation needs [5,7,8]. Field pollution monitors/sensors, required by the Environmental Protection Agency (EPA), are the primary method of measuring pollution for health advisories and pollutant reduction. Estimating ozone pollution is challenging as concentrations vary significantly in urban environments and fixed site monitoring is limited in predicting personal exposures [9].

Many sensor placement strategies do not consider population demographics (EPA, 2021), and consequently, air pollution networks may produce inequities and disproportionate health burdens on vulnerable populations [10,11]. Unfortunately, this practice conflicts with the National Ambient Air Quality Standards (NAAQS) and the Clean Air Act, as its regulatory framework contains provisions for the protection of sensitive groups, leading to an “adequate margin of safety for the entire population” [12,13]. Similarly, Miranda et al. (2011) evaluated the intent of the CAA in representing both advantaged and disadvantaged populations and found that race, age, and poverty demographics were significant indicators of increased pollution [14]. Consequently, low-income, Black communities experienced higher ambient pollution levels. The study also concluded that the current US pollution monitoring networks leave large portions of the population without exposure representation (monitoring data) [14].

The integration of a pro-vulnerability policy in designing air pollution networks can provide a more comprehensive approach to addressing inequities [15]. Consequentially, socially vulnerability theory suggests that adverse circumstances do not affect population groups uniformly [16,17]. Vulnerabilities are defined as an inability to protect oneself from different types of harm [18,19]; vulnerability is less about resources and more about power or powerlessness. The place vulnerability theory suggests that vulnerable people live in vulnerable places [20,21,22,23]. Using a vulnerability index in conjunction with other effects, such as urban heat islands, Sabrin et al. (2020) was able to target and prioritize areas with enormous social challenges and ozone and PM exposure [24]. Similarly, Wright and Diab (2011) developed a vulnerability-to-risk prioritization framework to ascertain at-risk communities and reduce pollution exposure impacts [25]. For air pollution, focusing on vulnerable populations is a necessary approach to public health protection [17,26], but it is missing from current air pollution–monitoring policy. We use the vulnerability framework as a tool to identify vulnerable populations that are at an increased risk of air pollution exposure.

In this paper, we investigate the associations and explore the measurement of the effect between areas of no data coverage (census tracts outside of sensor/monitor range) and the US Census vulnerability indicators in four themes from the Centers for Disease Control Agency for Toxic Substances and Disease Registry Social Vulnerability Index (CDC/ATSDR SVI): socioeconomic status, household characteristics, racial and ethnic minority status, and housing type/transportation. Consequently, the intent of this paper is to build knowledge about areas of no coverage and population social vulnerability characteristics, exploring whether the social vulnerability index (SVI) variables are significant effect modifiers of the relationship between no coverage and ozone air pollution.

## 2. Materials and Methods

Dallas–Fort Worth (DFW) was selected as the study area, comprising 10 counties, 1292 census tracts, and a population of over 7 million people [27]. It contains 17 regulatory ozone monitors, and the (9-county, not including Wise County) region is currently designated in noncompliance with the NAAQS for ozone [28]. This means that the region exceeds the regulatory standard of 70 parts per billion (2015), as the fourth-highest daily maximum 8-hour concentration, averaged across 3 consecutive years [28,29].

Continuous data to visualize the spatial patterns in Dallas–Fort Worth (DFW) are from the SVI created by the CDC/ATSDR at the census tract level [27]. The SVI uses the American Community Survey [30] 5-year data (2014–2018) and ranks each tract on 15 social factors grouped into 4 themes: socioeconomic status, household composition, race/ethnicity/language, and housing/transportation. The SVI also contains dichotomized data identifying the 90th-percentile or the most vulnerable populations within each social factor. The decision-making structure for the evaluation of populations covered or not covered by air pollution sensors in DFW from Northeim, Oppong, and Tiwari (2021) is applied to the spatial scales (service radii) of 4, 6, 10, and 20 km in the DFW region [31,32].

First, we use the independent samples *t*-test (IBM SPSS Statistics 27) to evaluate the difference in population social vulnerability continuous variables inside and outside each spatial scale. Outside the spatial scale is deemed “uncovered, outside coverage”, and conversely, inside the spatial scale is “covered, inside coverage”. The goal is to assess whether the social vulnerability characteristics of those within the coverage distance are different from those outside. Next, a geospatial analysis and visualization of the SVI throughout DFW is performed using the standard deviation classification scheme on ESRI ArcMap 10.7.1/ArcGIS Pro 2020. This method reduces bias without adding more classes and made a consistent comparison to the independent samples *t*-test. We use choropleth maps for spatial pattern visualization.

Then, to examine the risk measurement of the effect, we perform a multinomial logistic regression for classification and predictive analysis for the 10 and 20 km spatial scales because they most closely represent actual sensor coverage in DFW. As in Northeim, Tiwari, and Oppong (2021), Voronoi catchment areas and proximal allocation heuristic modeling are used to estimate and categorize the regions and populations served by the existing monitoring network [31]. Finally, using the CDC/ATSDR SVI dichotomized data, we examine all 15 variables to identify the 90th percentile and calculate the odds ratio as a measure of association between the exposure and the outcome [33,34]. These 90th-percentile values are the census tracts in the top 10% and are identified as the most vulnerable populations [35].

## 3. Results

Independent samples *t*-test were performed. Table 1 shows that at the 4 km spatial scale, the covered population was not statistically different from the uncovered population (*p* > 0.05). At the 6 km spatial scale, the covered and uncovered populations were significantly different (*p* < 0.05). The uncovered area had a higher presence of mobile homes, while per capita income, speaks English “less than well”, multiunit structures, and crowding were higher in the covered area. At the 10 km spatial scale, the covered and uncovered population means were also significantly different. The uncovered area had a higher presence of people living below poverty, no high school diploma, aged 65 or older, aged 17 or younger, older than age 5 with a disability, single-parent households, and mobile homes (Table 1). Similarly, per capita income and multiunit structures were higher in the covered area. Finally, at the 20 km spatial scale, the covered and uncovered population means were significantly different. The uncovered area had a higher presence of people living below poverty, no high school diploma, aged 65 or older, aged 17 or younger, older than age 5 with a disability, single-parent households, and mobile homes. Following a similar trend, per capita income, multiunit structures, and no vehicle were higher in the covered area.

**Figure 1:**
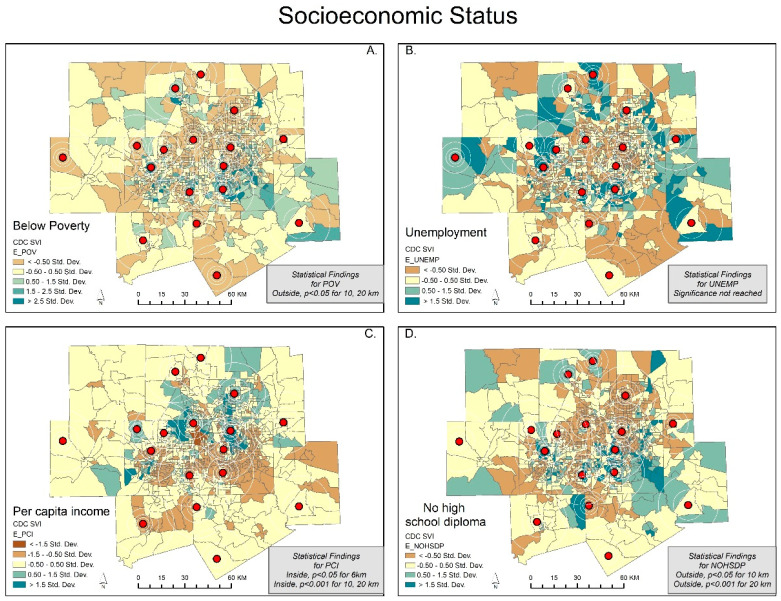
Dallas—Fort Worth: SD classification scheme of social variables for socioeconomic status. The CDC/ATSDR SVI variables include Figures; (**A**) below poverty; (**B**) unemployment; (**C**) per capita income; (**D**) no high school diploma.

**Figure 2:**
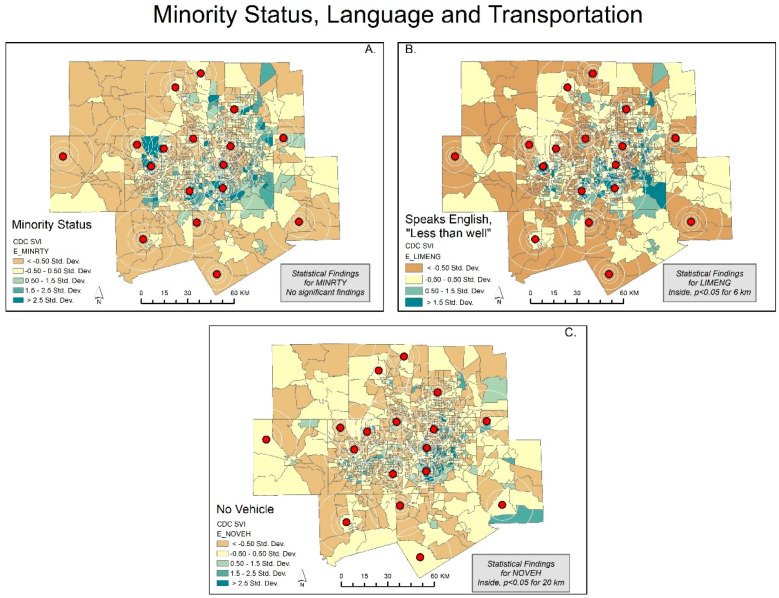
Dallas—Fort Worth: SD classification scheme of social variables for minority status, language and transportation. The CDC/ATSDR SVI variables include Figures; (**A**) minority status; (**B**) speaks English, “less than well”; (**C**) no vehicle.

**Figure 3:**
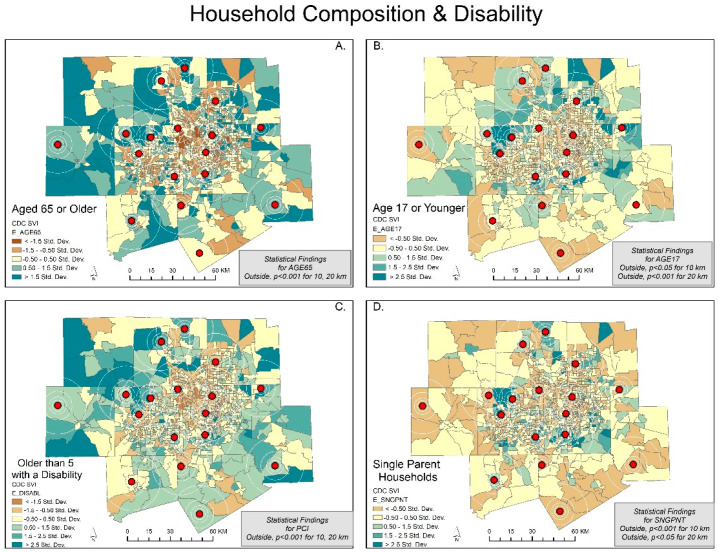
Dallas—Fort Worth: SD classification scheme of social variables for household composition and disability. The CDC/ATSDR SVI variables include Figures; (**A**) aged 65 or older; (**B**) age 17 or younger; (**C**) older than 5 with a disability; (**D**) single parent households.

**Figure 4:**
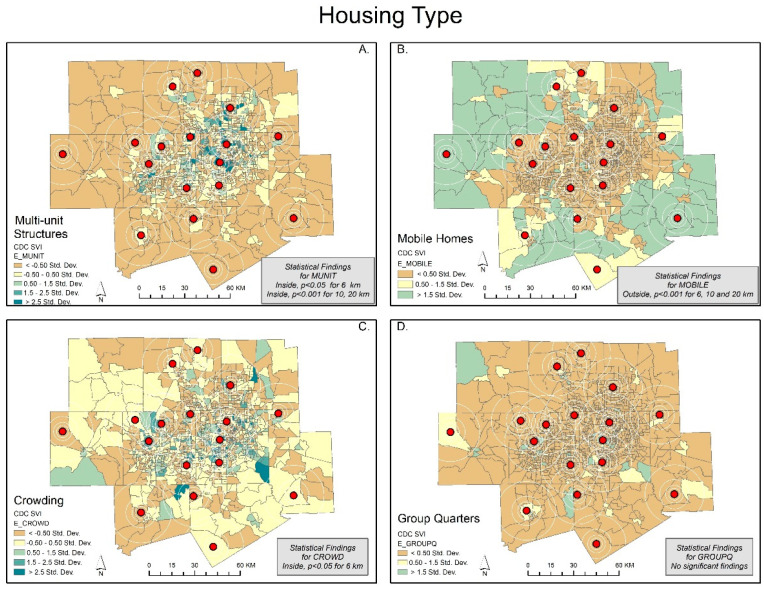
Dallas—Fort Worth: SD classification scheme of social variables for housing type. The CDC/ATSDR SVI variables include Figures; (**A**) multi-unit structures; (**B**) mobile homes; (**C**) crowding; (**D**) group quarters.

We show each map with its corresponding statistical findings in Figure 1, Figure 2, Figure 3 and Figure 4. The rings show the coverage distance (4, 6, 10, and 20 km service radius) for each of the SVI indicators, and the centroids represent the existing monitoring facility. For example, Figure 1A highlights the SVI variable of below poverty using five colored classifications. The mean of the independent samples *t*-test is significant and greater for the not covered or outside of the service radii for 10 and 20 km. Areas that are covered tend to have a lower percent below poverty. Meanwhile, areas that are not covered tend to have higher levels of people living below poverty. Large portions of DFW display orange and yellow census tracts with values lower than the standard deviation for below poverty (indicating less severity), while clusters in southeast Dallas, central and eastern Tarrant County, and outliers in all other counties show values greater than the standard deviation for below poverty (indicating more severity). This variable is the opposite of the per capita income variable (Figure 1C).

Table 2 displays the multivariate logistic regression Exp(B) odds ratios between the SVI 90th-percentile variables and the not-covered (outside) modality. The logit was performed in IBM SPSS version 27.

## 4. Discussion

As in Cutter et al. (2003), a social vulnerability index was created for this study to examine the spatial patterns of natural hazards at a county level in the US to understand more about at-risk populations. By identifying the locations of vulnerable populations and their proximity to air pollution sensors, we assert that such approaches reveal potential disproportionate exposure to ozone pollution and probably underestimate the exposure of the vulnerable. In addition, current ozone alerts may be irrelevant to those who live in such uncovered areas. Previous indicators of vulnerability in the literature vary depending on the method and the design of the study, although evidence continues to support the concept that air pollution networks designed without a vulnerability framework in mind introduce inherent biases and errors [36].

Current research suggests that underserved or vulnerable populations are more likely to experience premature death and serious health effects from exposure to pollution [1,37,38]. Simoni et al. (2015) reported that frailty and pre-existing diseases increase susceptibility to mortality caused by air pollution [39]. Thus, the elderly with chronic exposure to air pollution had higher incidences of chronic obstructive pulmonary disease (COPD), bronchitis, asthma, and emphysema [39]. Vulnerable populations are at risk of air pollution because large-scale disasters likely affect the health of these fragile communities [40,41]. Identifying factors and mapping social vulnerability are important pieces in environmental management [42]. 

Our *t*-test results show that the characteristics of the populations inside the spatial scale of coverage were statistically different from the populations outside the coverage areas. Clearly, socially vulnerable populations are less likely to be covered within the current sensor network. Out of the 15 SVI variables, only minority status, unemployment, and group quarters were not significantly different at the 6, 8, and 20 km scales. At the 4 km spatial scale, there is no significant difference in any of the variables. The maps support this result (Figure 1, Figure 2, Figure 3 and Figure 4).

Initially, we expected to find minority status to be a significant predictor of no coverage, but that is not the case (Figure 2A). Upon close inspection, Figure 2A shows that much of the population with minority status is concentrated in the Tarrant and Dallas counties. A small portion of the southeastern Dallas and southern Tarrant counties are not within the service radius (4, 6, 10, and 20 km), but owing to the vast number of nonminority clusters in the urban fringe, the difference was not significant.

The Census defines urbanized areas to have populations greater than 50,000 and urban clusters of at least 2500 but not greater than 50,000 [43]. Reporting social characteristics by census tract enables us to visualize urban or rural status, allowing for a deeper examination or interpretation of spatial patterns because the community structures are vastly different [44]. An interpretation of the delineation of geographical areas for SVI in DFW shows a potential pattern of urban versus rural place characteristics. For example, significant disparities already exist within the rural population owing to access to healthcare and a lack of resources [45,46], and in this case, there is spatial evidence, where urban air pollution influences could complicate rural social disparities.

We expected to find social variables that predicted pollution data coverage but found the place characteristics of the vulnerable to be more important. A clear example is Wise County, which is the only county in DFW without an ozone air pollution monitor/sensor. In fact, the county has 100% no coverage until the service radius reaches 10 km (coverage less than 5%) and 20 km (coverage less than 25%) (Figure 1, Figure 2, Figure 3 and Figure 4) [31]. Wise County is in the northwest corner of DFW and most likely has the largest ozone exposures [47,48].

In the logit regression analysis, the variable that predicted no coverage was mobile homes (OR = 4.831, 95% CI [2.500–9.338] and OR = 8.066, 95% CI [4.390–14.820] for the 10 and 20 km spatial units, respectively). Moreover, this may indicate that established neighborhoods or select parts of DFW are more likely to have coverage, whereas newer areas, outside the population center, are less likely to have coverage. Consequentially, this raises questions whether the current placement policy adequately accommodates population increases or new developments. The variables that predicted coverage most frequently were multiunit structures (no coverage, OR = 0.281, 95% CI [0.281–0.548] and OR = 0.130, 95% CI [0.037, 0.457] for the 10 and 20 km spatial units, respectively) and speaks English “less than well” (no coverage, OR = 0.521, 95% CI [0.292–0.931] for 10 km). The speaks English “less than well” clustering in Figure 4A. shows much of the population in the urban center, covered by air pollution centers. The multiunit structures result is consistent with expectations in that most of the multiunit structures are in highly populated environments (including downtown), and this is true in DFW (Figure 4A). We conclude that there are spatial variations in coverage characteristics that leave some highly vulnerable mobile home populations at high risk to levels of unknown air pollution.

## 5. Conclusions

We investigated the relationship between socially vulnerable populations and ozone air pollution–monitoring coverage in DFW. Areas with a higher concentration of mobile homes were a significant effect modifier of the relationship between no coverage and ozone air pollution and were more likely to have no coverage. Air pollution–monitoring policy needs to take proper measures to address this and other potential imbalances to ensure reliable and equitable air quality–forecasting predictions for all places, including those occupied by mobile homes. In fact, because of the well-known impact of air pollution on human health, especially for vulnerable populations, social vulnerability characteristics should be explicitly considered in future air pollution–monitoring sensor placement. Our results provide insights for optimizing sensor placements in the future to ensure equitable pollutant control for all.

## Figures and Tables

**Table 1:** Independent samples *t*-test on SVI variables by spatial scale.

Scale	Not Covered = Outside Spatial Scale		Covered = Inside Spatial Scale	
	Variable	Description	Variable	Description
4 km	–	not significant	–	not significant
6 km	E_MOBILE **	Mobile homes	E_PCI *	Per capita income
			E_LIMENG *	Speaks English “less than well”
			E_MUNIT *	Multiunit structures
			E_CROWD *	Crowding
10 km	E_POV *	Below Poverty	E_PCI **	Per capita income
	E_NOHSDP *	No High School Diploma	E_MUNIT **	Multiunit structures
	E_AGE65 **	Aged 65 or Older		
	E_AGE17 *	Aged 17 or Younger		
	E_DISABL **	Older than Age 5 with a Disability		
	E_SNGPNT *	Single-Parent Households		
	E_MOBILE **	Mobile Homes		
20 km	E_POV *	Below Poverty	E_PCI **	Per capita income
	E_NOHSDP **	No High School Diploma	E_MUNIT **	Multiunit structures
	E_AGE65 **	Aged 65 or Older	E_NOVEH *	No Vehicle
	E_AGE17 **	Aged 17 or Younger		
	E_DISABL **	Older than Age 5 with a Disability		
	E_SNGPNT *	Single-Parent Households		
	E_MOBILE **	Mobile Homes		

Note: * *p* < 0.05, ** *p* < 0.001. Dallas–Fort Worth group statistics at all spatial scales. Note: All effect sizes were small.

**Table 2:** Multinomial logistic regression of 90th-percentile SVI variables by spatial scale for not covered, on 15 individual variables (N = 1293 census tracts).

Variable	Description		Odds Ratio (95% CI)
–		10 km	20 km
F_POV	Below Poverty	0.563 (0.288–1.101)	0.619 (0.142–2.694)
F_UNEMP	Unemployment	0.847 (0.485–1.481)	0.585 (0.169–2.024)
F_PCI	Per Capita Income	1.753 (0.913–3.3640)	1.138 (0.377–3.437)
F_NOHSDP	No High School Diploma	0.958 (0.541–1.697)	1.715 (0.638–4.613)
F_AGE65	Aged 65 or Older	0.955 (0.576–1.583)	0.841 (0.304–2.326)
F_AGE17	Aged 17 or Younger	1.018 (0.679–1.528)	2.177 (1.159–4.092)
F_DISABL	Older than Age 5 with a Disability	0.717 (0.302–1.700)	2.189 (0.498–9.628)
F_SNGPNT	Single-Parent Households	1.886 (1.242–2.863)	2.094 (1.066–4.117)
F_MINRTY	Minority Status	0.939 (0.524–1.682)	0.493 (0.142–1.711)
F_LIMENG	Speaks English “Less Than Well”	0.521 (0.292–0.931)	0.762 (0.259–2.242)
F_MUNIT	Multiunit Structures	0.392 (0.281–0.548)	0.130 (0.037–0.457)
F_MOBILE	Mobile Homes	4.831 (2.500–9.338)	8.066 (4.390–14.820)
F_CROWD	Crowding	0.972 (0.609–1.549)	0.453 (0.174–1.176)
F_NOVEH	No Vehicle	1.467 (0.885–2.432)	0.367 (0.091–1.479)
F_GROUPQ	Group Quarters	0.723 (0.432–1.209)	2.593 (1.224–5.493)

Note: The reference modality is covered (2).

## Data Availability

Centers for Disease Control and Prevention/Agency for Toxic Substances and Disease Registry/Geospatial Research, Analysis, and Services Program. CDC/ATSDR Social Vulnerability Index [2018] Database [Texas].
